# Negative Modulation of the Angiogenic Cascade Induced by Allosteric Kinesin Eg5 Inhibitors in a Gastric Adenocarcinoma In Vitro Model

**DOI:** 10.3390/molecules27030957

**Published:** 2022-01-31

**Authors:** Alessia Ricci, Marialucia Gallorini, Donatella Del Bufalo, Amelia Cataldi, Ilaria D’Agostino, Simone Carradori, Susi Zara

**Affiliations:** 1 Department of Pharmacy, University “G. d’Annunzio” of Chieti-Pescara, 66100 Chieti, Italy; alessia.ricci@unich.it (A.R.); marialucia.gallorini@unich.it (M.G.); amelia.cataldi@unich.it (A.C.); ilaria.dagostino1@unich.it (I.D.); simone.carradori@unich.it (S.C.); 2 Preclinical Models and New Therapeutic Agents Unit, IRCCS Regina Elena National Cancer Institute, 00144 Rome, Italy; donatella.delbufalo@ifo.it

**Keywords:** kinesins, Eg5 inhibitors, gastric adenocarcinoma, hesperidin, angiogenesis, in silico studies

## Abstract

Eg5 is a kinesin essential in bipolar spindle formation, overexpressed in tumours, thus representing a new target in cancer therapy. We aimed at evaluating the anti-cancer activity of Eg5 thiadiazoline inhibitors **2** and **41** on gastric adenocarcinoma cells (AGS), focusing on the modulation of angiogenic signalling. Docking studies confirmed a similar interaction with Eg5 to that of the parent compound **K858**. Thiadiazolines were also tested in combination with Hesperidin (HSD). Cell cycle analysis reveals a reduction of G1 and S phase percentages when **41** is administered as well as HSD in combination with **K858**. Western blot reveals Eg5 inhibitors capability to reduce PI3K, p-AKT/Akt and p-Erk/Erk expressions; p-Akt/Akt ratio is even more decreased in HSD+**2** sample than the p-Erk/Erk ratio in HSD+**41** or **K858**. VEGF expression is reduced when HSD+**2** and HSD+**41** are administered with respect to compounds alone, after 72 h. ANGPT2 gene expression increases in cells treated with **41** and HSD+**2** compared to **K858**. The wound-healing assay highlights a reduction in the cut in HSD+**2** sample compared to **2** and HSD. Thus, Eg5 inhibitors appear to modulate angiogenic signalling by controlling VEGF activity even better if combined with HSD. Overall, Eg5 inhibitors can represent a promising starting point to develop innovative anti-cancer strategies.

## 1. Introduction

Gastric cancer is the fifth most frequent type of cancer and deadly disease diagnosed worldwide. It was responsible for 1,000,000 new cases of cancer (with an incidence of 5.7%) and approximately 783,000 deaths (8.2%) in 2018. Men have a two-fold higher rate of mortality than women, with an incidence of 7.2% and 9.2%, respectively, mainly in Asian countries [1]. There are three main subtypes of gastric cancer, historically classified from 1960 by Lauren’s histological classification, which divide gastric cancer into well-differentiated subtype (intestinal subtype), poorly differentiated subtype (diffuse subtype), and mixed disease (when both intestinal and diffuse types are present) [2]. Several causes can trigger gastric cancer development, among them, pathogenic infections such as *Helicobacter pylori*, which represents 90% of new gastric cancer cases, [3] and Epstein–Barr virus; non-pathogenic causes (familial and hereditary conditions, smoking, diet, alcohol, etc.), sedentary lifestyle, and others are included [4,5]. Considering that prevention is the most important approach, there is not a standard therapy to treat gastric cancer; thus, complete surgical resection remains the best option to cure patients. To improve prognosis, usually, a combination of surgery and chemotherapy or radiotherapy is applied. Common treatment protocols include the administration of epirubicin, cisplatin, 5-fluorouracil, oxaliplatin, and docetaxel, as well as targeted therapy with monoclonal antibodies or small inhibitor molecules [4,6,7]. The discovery of novel treatment options for gastric cancer to improve patient survival still represents a crucial point.

The kinesins superfamily (KIFs) are motor proteins that are present in eukaryotes and are able to use the energy obtained from the ATP hydrolysis into their motor domain to allow movements along the microtubules and perform different and essential activities into the cells, such as intracellular vesicle and organelle transport, bipolar spindle assembly, microtubule remodelling, and neuronal plasticity. Almost 650 members were identified and classified in approximately 14 subfamilies: each member belongs to a specific subfamily based on structural and functional properties [8,9]. All kinesins present a motor domain named “head”, with high grade retained within families, a globular tail, and a junction region between head and tail, named the stalk. The head motor domain has a specific structure designed to allow a cyclic attachment-detachment from microtubules, due to the energy obtained from ATP hydrolysis [10], and it is located differently in each subfamily. It is present in the *N*-terminal or *C*-terminal region or in the middle of the protein, in N-kinesins, C-kinesins, and M-kinesins, respectively. Based on their activities, there are mitotic kinesins and non-mitotic kinesins (neuronal, organelle, and vesicle transport, ciliary kinesins) [11]. Over the last years, the involvement of kinesins in the genesis and development of several tumours has become clear. Both kinesins and their inhibitors are gaining greater interest in the last twenty years, as demonstrated by the intense research activity of the scientific community (Figure S1). Furthermore, the phylogenetic analysis of human kinesins, obtained by comparing the reviewed protein sequences present in the UniProt Database, seems to confirm their applicability as promising pharmacological targets in drug discovery programmes [12] and the development of selective inhibitors. The dendrogram reported in Figure S2 highlights large differences among certain enzymes in the superfamily, especially in some cases.

**Figure 1:**
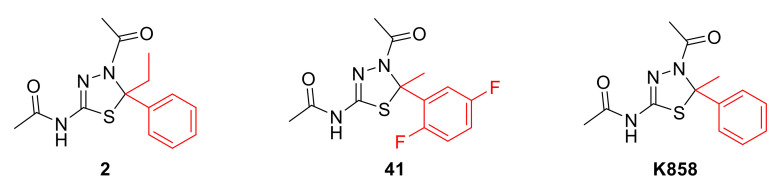
Chemical structures of Eg5 inhibitors **2** and **41** and their parent compound **K858**. In red, chemical modifications in position 5 of the thiadiazoline core are highlighted.

As proof, KIF2A and KIF20A were found to have a prognostic significance in different types of cancer [13], and other kinesins subfamilies play a role in breast cancer [14], osteosarcoma [15], colorectal cancer [16], and many others. In particular, KIF11, known as Eg5 or kinesin spindle protein (KSP), being overexpressed in gastric [17], breast [18], pancreatic [19], prostate [20], and bladder cancers [21] and glioblastoma [22], is worthy of in-depth investigation. KIF11 is a N-kinesin microtubule plus-end-directed motor protein essential during mitotic bipolar spindle formation. When its activity is inhibited, the bipolar spindle fails to assemble and a monopolar spindle forms, thus resulting in cell death. Based on these findings, different Eg5 inhibitors were synthesized and tested in in vitro cancer models, and the most promising have entered clinical trials [23,24,25,26], such as the dihydropyrimidine scaffold-based [27,28], quinazolinones [26], chromen-4-ones [29], benzimidazoles [30], and thiadiazolines [31]. Some inhibitors are also highlighted in the protein–chemical network maps obtained through the STITCH tool [32] and reported in Supporting Information in Figure S3. Among them, **K858**, a substituted 1,3,4-thiadiazoline endowed with an asymmetric C5 atom (Figure 1), displayed a remarkable inhibitory activity profile [33].

**Figure 2:**
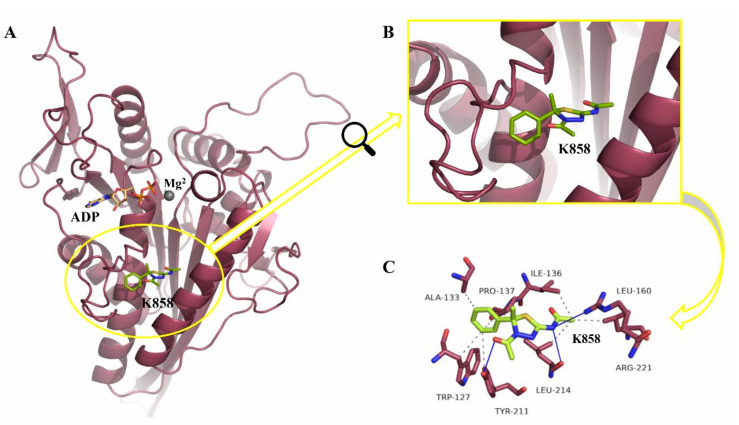
Structure of the **K858**-Eg5-ADP complex and zoom on interaction residues. (**A**) Enzyme–ligand complex. The cocrystal structure is from PDB (entry code: 6G6Z), in space groups P6522. The overall structure of the Eg5 motor domain (red cartoon) with Mg^2+^ (grey sphere) and ADP (yellow sticks) is bound in the nucleotide-binding pocket. (*S*)-**K858** (lime sticks), the only active enantiomer of the racemic **K858**, is located in the allosteric inhibitor-binding pocket. (**B**) Zoom on the binding site of **K858**. (**C**) Interaction residues and **K858**. Hydrogen bondings are shown in blue solid lines. Hydrophobic contacts and shown in grey dotted lines. The figure was prepared through Protein-Ligand Interaction Profiler (PLIP) web service [34] and the PyMOL Molecular Graphics System, Version 2.5 Schrödinger, LLC [35].

This compound is able to selectively inhibit the Eg5 ATPase activity in an ATP-uncompetitive manner with an IC_50_ of 1.3 μM, being 150-fold more potent against Eg5 than other mitotic kinesins, such as CENP-E and MKLP1, and the kinesin heavy chain. The **K858**-Eg5-ADP cocrystal structure [33] is provided in Figure 2.

It provokes mitotic arrest accompanied by monopolar spindles, with a non-neurotoxic behaviour. In addition, **K858** was found to induce cancer cell death in vitro and in vivo [25]. We recently developed a large library of structural analogues of **K858**, maintaining the heterocyclic core and modifying the substitutions in C5. Among them, seven thiadiazolines emerged for their promising in vitro inhibition of the Eg5 ATPase activity with IC_50_ values ranging from 0.84 to 7.5 µM [36] and were further tested on gastric adenocarcinoma AGS cell line, resulting as a valuable strategy to both counteract tumour proliferation and invasiveness in monotherapy and activating the apoptotic cascade activation in combination with **K858**. In particular, compounds **2** and **41**, bearing an ethyl instead of a methyl substituent and a 2,5-difluorophenyl group in lieu of the unsubstituted phenyl ring with respect of **K858** (depicted in Figure 1) emerged for their notable biological activity at a concentration of 1 and 5 µM, respectively, becoming attractive for a perspective in-depth investigation in gastric adenocarcinoma cell line [37].

Moreover, protein-protein association network maps, obtained through the STRING tool [38] and reported in Supporting Information in Figure S4, show that Eg5 interacts with several proteins involved in gastric cancer, such as (TUBA1B), Tubulin beta-2B chain (TUBB2B), Disks large-associated protein 5 (DLGAP5), microtubule nucleation factor (TPX2), Dynactin subunit 1 (DCTN1), and the well-known kinases AURKA and CDK1, confirming its involvement in multiple pathways and clinical outcomes of the disease (see Supporting Information for references).

As widely known, a diet rich in fruit and vegetables could counteract the onset of some types of tumours due to the appreciable content of anti-inflammatory, antioxidant, antiproliferative, and pro-apoptotic polyphenols [39]. It has been demonstrated that Hesperidin (HSD), the most abundant polyphenol in citrus fruits, has a valuable antiproliferative and antitumour effect on different cancer cell lines due to its ability to induce a cell cycle arrest and apoptosis [40]. Moreover, in some studies, HSD was administered in combination with well-known anti-cancer drugs, such as doxorubicin, 5-fluorouracil, or tamoxifen, to obtain a synergistic effect [41]. Thus, the current work is aimed at investigating the potential antitumoral effect of two previously reported Eg5 inhibitors, **2** and **41** [37], with respect to their parent compound **K858** on the AGS cell line. In particular, their antiproliferative effect and the capability in controlling angiogenic pathways were assessed. In addition, they were also tested in combination with HSD.

## 2. Results

### 2.1. Docking Simulation Studies

**Figure 3:**
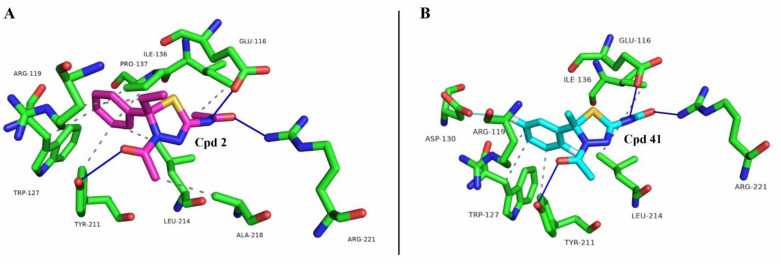
Docking pose of compounds **2** (**A**) and **41** (**B**) with Eg5. Interacting-residues are represented by green sticks and labelled. Compounds **2** and **41** are presented in purple and cyan sticks, respectively. Hydrogen bondings and hydrophobic contacts are shown in solid blue and dotted grey lines, respectively. Halogen bonding is reported through a solid cyan line. The figure was prepared through Protein-Ligand Interaction Profiler (PLIP) web service [34] and the PyMOL Molecular Graphics System, Version 2.5 Schrödinger, LLC [35].

First, a docking simulation on compounds **2** and **41** was performed to define their interactions with Eg5 (Figure 3).

The computational analysis confirmed that both the compounds fit the binding site with a similar pose of **K858** (see Figure 2C and Figure 3) and establish a complex hydrogen-bonding network, although with different residues. Derivatives **2** and **41** interact with the phenol moiety of Tyr211 through the acetyl function on the thiadiazoline ring, while two additional hydrogen bonds are formed by the carbonyl group and the nitrogen atom in C2 with the guanidino tail of Arg221 and the carboxylic function of Glu116, respectively. Meanwhile, **K858** interacts in the same fashion with Tyr211, while its nitrogen in C2 forms hydrogen bondings with Arg221 and Leu214. As regards the hydrophobic contacts, although placed into the same site with good superposition, the small changes in their chemical structures seem to highlight a different residue interaction network. PLIP analysis indicates that the compounds generate hydrophobic interactions with Trp127, Ile136, Tyr211, and Leu214. Moreover, **K858** establishes hydrophobic contacts with Ala133, Pro137, and Leu160 (Figure 2C), while compound **2** with Arg119, Pro137, and Ala218 (Figure 3A). Instead, compound **41** can form a hydrophobic interaction with Arg119 and, also, a halogen bond with Asp130 through the fluorine atom on the phenyl ring in *meta* position with respect to the thiadiazoline ring (Figure 3B).

### 2.2. Effect of HSD and Eg5 Inhibitors on AGS Cell Proliferation

**Figure 4:**
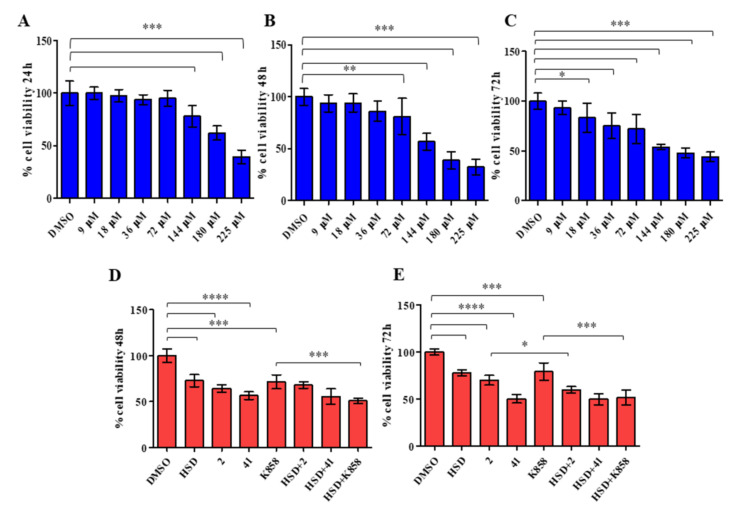
MTT test on AGS cell line treated with increasing doses of HSD (from 9 to 225 μM) for 24, 48, and 72 h (**A**–**C**, respectively). *** *p* < 0.0001; ** *p* < 0.001; * *p* < 0.01. MTT test on AGS cells treated with HSD (180 μM), **2** (1 μM), **41** (5 μM), **K858** (1 μM), and a combination of HSD with each Eg5 inhibitor for 48 and 72 h (**D**–**E**, respectively). **** *p* < 0.0005, *** 0.005 < *p* < 0.001, * *p* < 0.05. Metabolic activity was normalized to control cells treated with DMSO (0.5% as final concentration). DMSO: control vehicle. Data shown represent the mean ± SD of three independent experiments.

The effect of HSD on AGS cells viability was tested through the MTT assay. AGS cells were treated with HSD at concentrations ranging from 9 to 225 µM for 24, 48, and 72 h (Figure 4).

After 24 h of treatment with concentrations from 9 to 72 µM AGS cells viability is similar to control (DMSO); starting from 144 µM a significant decrease in cell viability, compared to control is recorded (Figure 4A). After 48 h of HSD administration, a statistically significant reduction in cell viability with respect to control is appreciable from 72 to 225 µM in a dose-dependent manner (Figure 4B). When the AGS cell line is treated with HSD for 72 h, a valuable and significant reduction in cell viability is recorded after treatment at 18 µM (Figure 4C). Based on these results, 180 µM HSD for 48 or 72 h was chosen for further investigations considering that it provokes a cell viability reduction of approximately 40–50%.

Then, the MTT test was carried out administering HSD and the Eg5 inhibitors **2**, **41,** and **K858**, previously selected and tested on AGS cell line [37]. AGS cells were treated with HSD at 180 µM, **2** and K858 at 1 µM, and **41** at 5 µM and with a combination of HSD 180 µM and Eg5 inhibitors, for 48 and 72 h (Figure 4D,E), as previously determined. At both time points, all compounds alone induce a statistically significant reduction in AGS cells metabolic activity compared with control, with a major extent for **41**. After 48 h of treatment, the combination HSD+**K858** induces a significant reduction in cell viability compared to **K858** alone (Figure 2D). After 72 h, the latter combination is confirmed to induce a significant decrease in cell metabolic activity with respect to **K858** alone, along with the combination HSD+**2** which statistically affects AGS cell line viability compared to **2** alone (Figure 4E).

### 2.3. Cell Cycle Modulation by Eg5 Inhibitors Alone and in Combination with HSD

**Figure 5:**
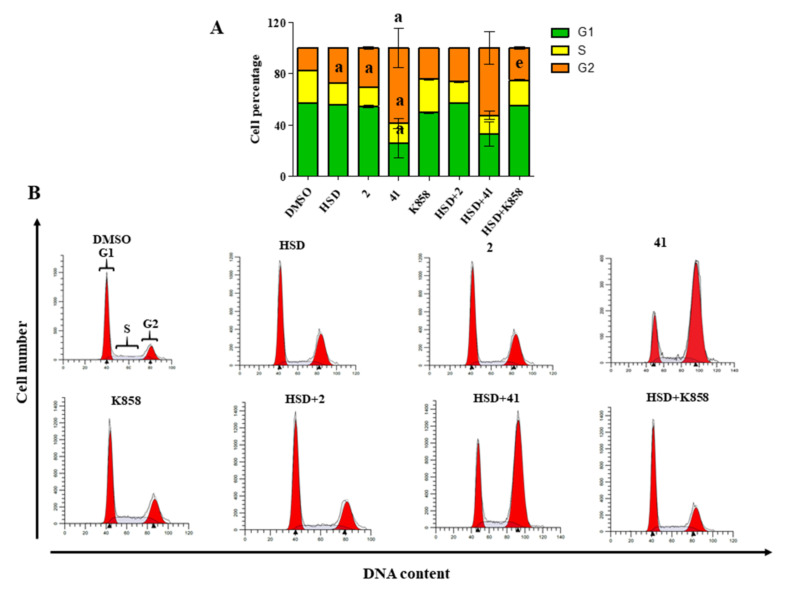
Cell cycle analysis in AGS cells treated with HSD (180 μM), **2** (1 μM), **41** (5 μM), **K858** (1 μM), and a combination of HSD with Eg5 inhibitors for 48 h. Data shown are mean ± SD of three independent experiments. (**A**) The bar graph shows cell percentages in the phases of cell cycle (G1, S, and G2) of AGS. DMSO 0.5%: control vehicle. a = *p* < 0.0001 between Eg5 inhibitors alone and DMSO; e = *p* < 0.001 between **K858** alone and HSD+**K858** combination. (**B**) Cell cycle profiles represented by fluorescence emission peaks obtained after propidium iodide staining (*y*-axis = cell count; *x*-axis = propidium iodide fluorescence emission in the FL- channel directly proportional with DNA content).

In order to explore the cause of AGS cells death, provoked by **2**, **41,** and **K858**, alone or in combination with HSD, a cell cycle analysis after 48 h of treatment was carried out (Figure 5A).

First, untreated cells (DMSO) show a standard cell cycle, being actively proliferative (G1 phase = 57%; S phase = 25.28%; G2 phase = 17.67%) (Figure 5B). When **41** is administered, an appreciable cell cycle modulation is recordable, for instance, G1 and S phase percentages are significantly reduced compared to the ones of the DMSO control (G1 = 25.54% and S = 15.60%, respectively), while the G2 phase cell percentage is significantly and strongly increased (58.85%), revealing the accumulation of treated cells in the G2 phase and thus a block in the latest stage of the cell cycle (Figure 5B). AGS cell line treated with **2** shows a weaker but significant decrease in the S phase with respect to control (14.91%) and consequently an accumulation of the cell population in the G2 phase, even if it does not appear statistically significant. Next, **K858** does not induce a statistically significant change in the percentages of cell population found in the various phases of the cell cycle compared to untreated cells. Lastly, cells were treated with HSD 180 µM and with a combination of HSD and Eg5 inhibitors after 48 h. HSD alone is able to significantly reduce AGS cells percentage found in the S phase with respect to control (17.26%) (Figure 5B), while, in combination with **K858**, it further significantly reduces the S phase percentage (19.92%) compared to that of **K858** alone, highlighting an accumulation in G1 phase (25.83%) (Figure 5B).

### 2.4. Effect of Eg5 Inhibitors in Monotherapy and Combination with HSD on VEGF Activity and PI3K/Akt Pathway

To evaluate the possibility of the new Eg5 inhibitors to modulate the angiogenic molecular cascades, we studied the modulation of PI3K/Akt pathway and VEGF expression and secretion in the AGS cell line.

**Figure 6:**
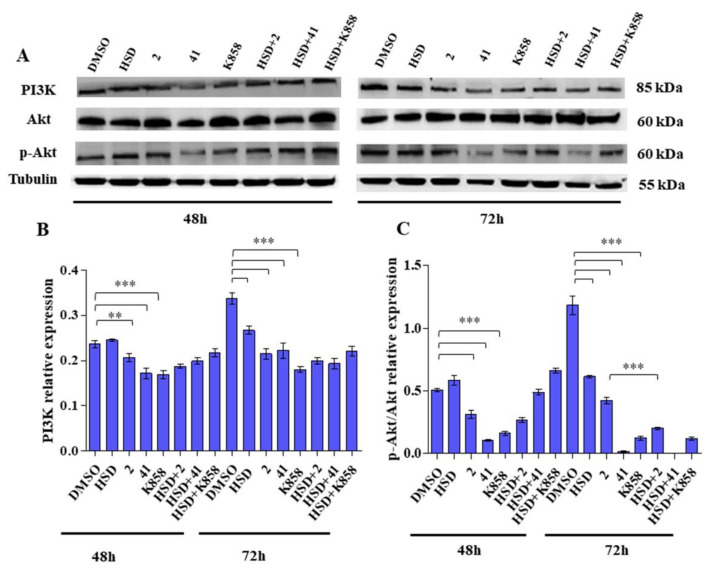
(**A**) PI3K and AKT expression levels in AGS cell line treated with HSD (180 μM), **2** (1 μM), **41** (5 μM), **K858** (1 μM), and a combination of HSD with each Eg5 inhibitor for 48 and 72 h. DMSO 0.5%: control vehicle. Data are reported as mean ± SD of three independent experiments. Tubulin is used as a loading control. (**B**,**C**) The bar graph displays densitometric values expressed as mean ± SD normalized on loading control. *** *p* < 0.0001; ** *p* < 0.001.

Western blot analysis revealed that after 48 h of treatment all the tested Eg5 inhibitors reduce PI3K protein expression with respect to the control. In particular, this reduction was more marked when AGS cells are treated with **41** and **K858**. After 72 h of treatment, PI3K expression shows the same trend, with a statistically significant reduction for all the three molecules with respect to control; in addition, HSD is able to significantly decrease PI3K levels (Figure 6B).

Western blot analysis for Akt protein, after 48 and 72 h of treatment, reveals a statistically significant reduction of p-Akt/Akt ratio compared to control in AGS cells treated with **2**, **41,** and **K858** after 48 h of culture, after 72 h this reduction is confirmed; HSD alone also induces a statistically significant decrease of p-Akt/Akt ratio. Additionally, after 72 h, HSD combined with **2** provokes a marked and statistically significant reduction in p-Akt/Akt ratio compared with the **2** alone (Figure 6C).

**Figure 7:**
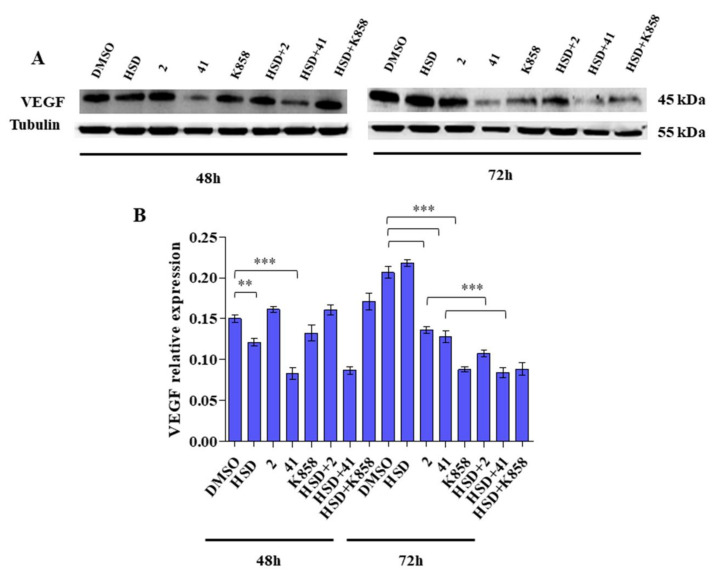
(**A**) VEGF expression levels in AGS cell line treated with HSD (180 μM), **2** (1 μM), **41** (5 μM), **K858** (1 μM), and a combination of HSD with each Eg5 inhibitor for 48 and 72 h. DMSO 0.5%: control vehicle. Data are reported as mean ± SD of three independent experiments. Tubulin is used as a loading control. (**B**) The bar graph displays densitometric values expressed as mean ± SD normalized on loading control. *** *p* < 0.0001; ** *p* < 0.001.

Western blot analysis of VEGF reveals that, after 48 h of treatment, HSD and **41** in monotherapies induce a significant reduction in VEGF expression, compared with control, while after 72 h, all the tested Eg5 inhibitors significantly decrease VEGF expression, respect to untreated cells. Moreover, at this time point, the combinations HSD+**2** and HSD+**41** significantly reduce VEGF expression with respect to compounds alone, thus enhancing the effect of the molecules (Figure 7).

**Figure 8:**
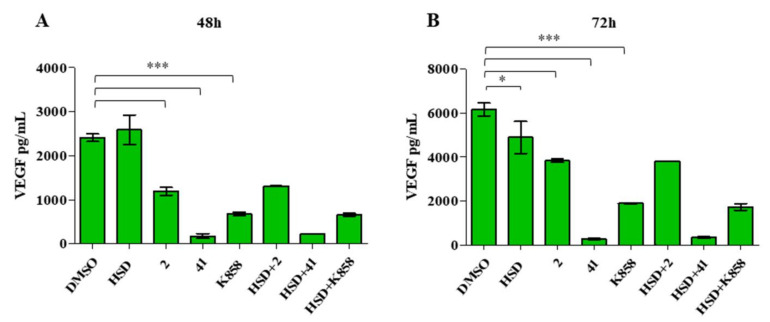
ELISA assay of AGS VEGF secretion. Cells were treated with HSD (180 μM), **2** (1 μM), **41** (5 μM), **K858** (1 μM), and a combination of HSD with each Eg5 inhibitor for 48 (**A**) and 72 h (**B**). Secretion levels are reported as pg/mL. The results are the mean ± SD of three samples from three different experiments. *** *p* < 0.0001; * *p* < 0.01.

ELISA analysis of VEGF release within the culture medium reveals that **2**, **41,** and **K858** reduce VEGF release with respect to the control (Figure 8A) after 48 and 72 h of treatment. Moreover, after 72 h of treatment, HSD significantly reduces VEGF release within the supernatant (Figure 8B).

### 2.5. Effect of Eg5 Inhibitors and HSD on Erk 1/2 Activation

**Figure 9:**
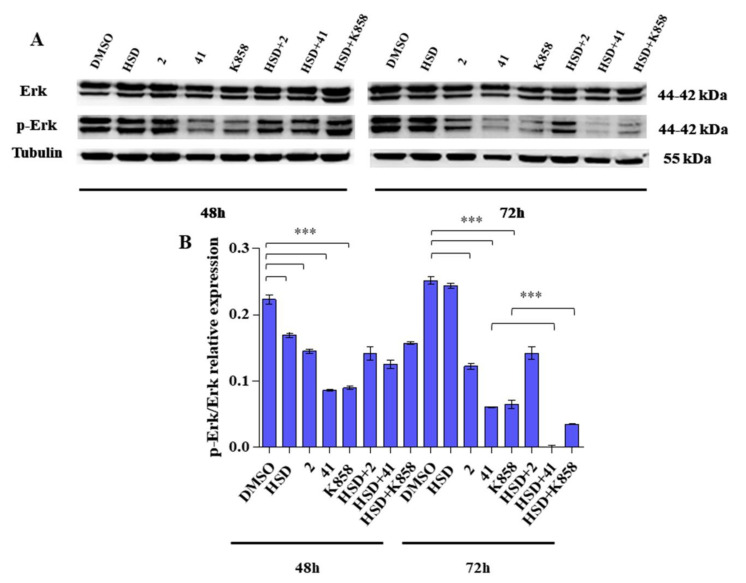
(**A**) Anti-p44/42 MAPK (Erk 1/2) and anti-phospho-p44/42 MAPK (p-Erk 1/2) expression levels in AGS cell line treated with HSD (180 μM), **2** (1 μM), **41** (5 μM), **K858** (1 μM), and a combination of HSD with each Eg5 inhibitor for 48 and 72 h. DMSO 0.5%: control vehicle. Data are reported as means ± SD of three independent experiments. Tubulin is used as a loading control. (**B**) The bar graph displays densitometric values expressed as mean ± SD of p-Erk/Erk ratio normalized on loading control. *** *p* < 0.0001.

To identify the effects of **2**, **41,** and **K858** on the activation of a specific MAPK, a Western blot analysis of Erk 1/2 was performed. After 48 and 72 h of treatment, all the tested Eg5 inhibitors determine a significant reduction in p-Erk/Erk ratio with respect to control. HSD is able to significantly reduce this ratio after 48 h of administration. In addition, after 72 h of treatment, HSD+**41** and HSD+**K858** combinations, significantly decrease p-Erk**/**Erk ratio with respect to **41** and **K858** alone (Figure 9B).

### 2.6. Modulation of ANGPT2 Gene Expression after Treatment with Eg5 Inhibitors Alone and in Combination with HSD

**Figure 10:**
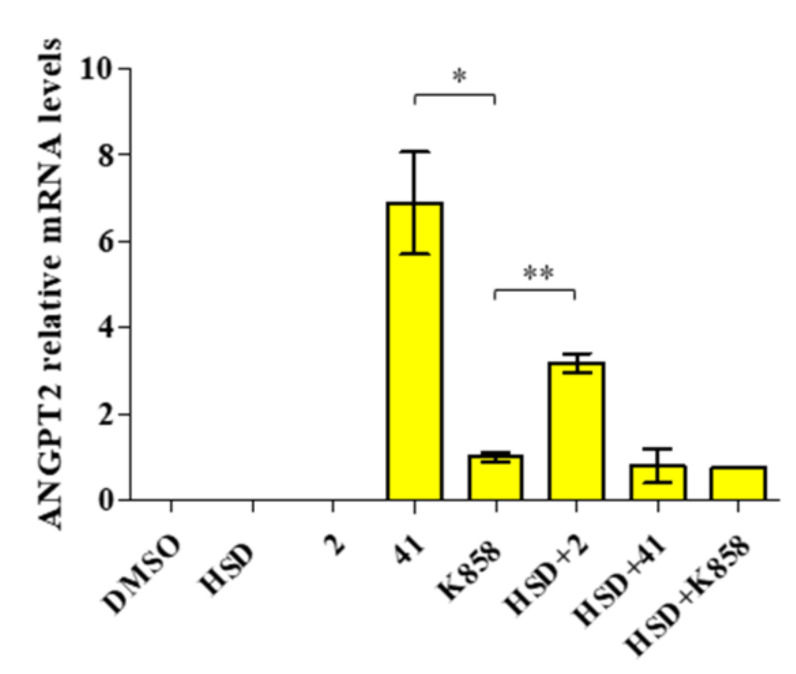
Relative gene expression of ANGPT2 in AGS cells treated with HSD (180 μM), **2** (1 μM), **41** (5 μM), **K858** (1 μM), and a combination of HSD with each Eg5 inhibitor for 24 h. Data are expressed as relative to **K858** as control (calibrator sample, defined as 1). Values represent means ± SD of three independent experiments. *Y*-axis, fold change. The most representative of three separate experiments is shown. ** *p* < 0.01; * *p* < 0.05.

ANGPT2 gene expression in AGS cells treated with **2**, **41,** and **K858**, alone and in combination with HSD was also investigated (Figure 10).

ANGPT2 is not expressed in untreated cells and AGS cells treated with **2** and HSD. A significant increase in gene expression level is detected in the AGS cell line treated with **41** compared with **K858**. The combination HSD+**2** shows a statically significant increase in ANGPT2 gene expression with respect to parent compound **K858**.

### 2.7. Effect of 2 in Combination with HSD on AGS Cells Migration

**Figure 11:**
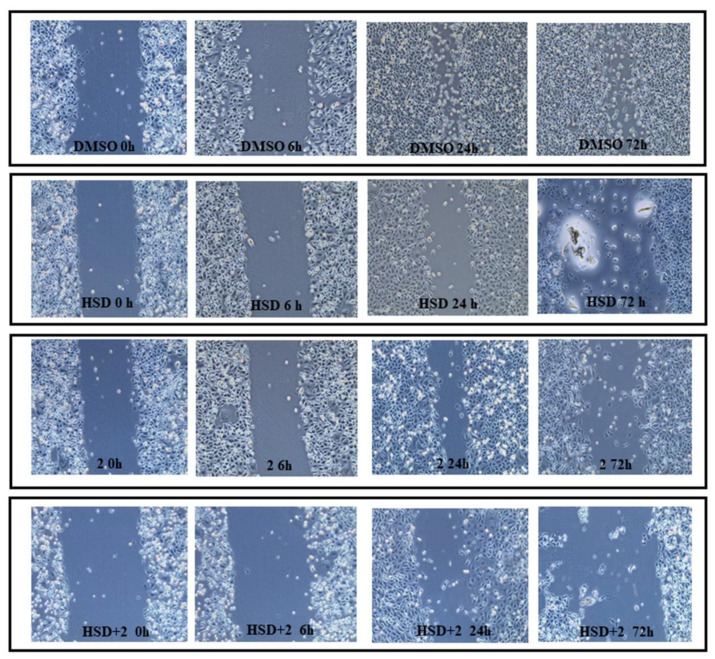
Scratch wound-healing assay was performed in AGS cells treated with HSD (180 μM), **2** (1 μM), and HSD+**2**. Images were taken at 0, 6, 24, and 72 h after confluent monolayer of cells was wounded. DMSO 0.5%: control vehicle. The most representative of three separate experiments is shown.

**Figure 12:**
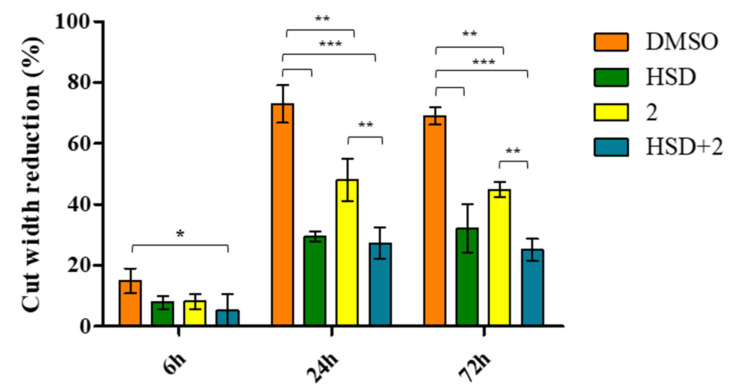
The bar graph displays the cut width reduction % obtained from scratch wound-healing assay in AGS treated with HSD (180 μM), **2** (1 μM), and HSD+**2** after 6, 24 and 72 h. DMSO 0.5%: control vehicle. *** *p* < 0.0001; ** *p* < 0.001; * *p* ≤ 0.05.

To evaluate the effect of **2** and HSD on AGS cell line migration, a wound-healing assay after 0, 6, 24, and 72 h, was performed. The cell monolayer scrape after 0 h of treatment is assumed as our starting point. After 6 h of treatment, there is a significant reduction of the cut width in AGS treated with HSD+**2** if compared with DMSO (DMSO = 14.91%; HSD = 7.88%; **2** = 8.11%; HSD+**2** = 5.18%). After 24 h of treatment, untreated AGS cells disclose a cut almost completely filled, with a width reduction of 72.98%, indicating that the AGS cell line has strong migratory potential, and this is significantly reduced with respect to all other experimental points (HSD = 29.42%; **2** = 48%; HSD+**2** = 27.28%). Moreover, cells treated with **2** reveal a statistically significant decrease in cut width compared to cells exposed to the combination HSD+**2**. A parallel trend is recorded after 72 h of treatment (DMSO = 69%; HSD = 32%; **2** = 44.91%; HSD+**2** = 25%) (Figure 11 and Figure 12).

## 3. Discussion

In the last two decades, researchers concentrated their efforts on the study of Eg5 and its value as a pharmacological target. In particular, the rise in interest in this protein is related to the discovery of novel drugs able to suppress its activity in tumours characterized by kinesin overexpression and, with this strategy, indirectly target the mitotic spindle, which is one of the most important goals of chemotherapy.

In previous work, we tested seven non-competitive Eg5 inhibitors analogues of the well-known **K858**, on the AGS cell line, and we selected two promising compounds, **2** and **41**, for further investigations. The ternary Eg5-ADP-**K858** complex, blocking the motor in the so-called final inhibitor bound state and halting ADP release, identified **K858** as an allosteric inhibitor to the binding pocket formed of helix α2, loop L5, and helix α3 [33]. Thiadiazolines **2** and **41** resulted in reduced AGS cells proliferation and migration, induce monoaster formation, and trigger apoptotic cascade, especially when combined with **K858** [26].

Polyphenol extracts from fruits and vegetables are also studied for their anti-cancer potential. HSD, a flavanone found in large amounts in citrus fruits, is a new compound tested in vitro on different cancer cell lines where it induced inhibition of cell proliferation by blocking the cell cycle through the reduction of apoptosis [40]. In addition, HSD, often administered in combination with well-known anti-cancer drugs, such as doxorubicin, 5-fluorouracil, or tamoxifen [41,42], can improve their cytotoxicity, increasing cancer cells sensitivity to chemotherapy. Thus, the aim of the present work is the evaluation of the effect of HSD on AGS cells, also estimating a possible improvement of its effect when combined with Eg5 inhibitors.

The cell cycle is a key cell process strictly regulated involving specific proteins such as cyclins and cyclin-dependent kinases which, translocating within the nucleus, regulate the beginning of the mitotic process or are required to trigger the G2/M transition [43,44].

As already demonstrated [37], **2** and **41** inhibit Eg5 activity inducing monoaster formation which, in turn, blocks mitotic division and prevents cells to move on M phase, provoking accumulation in G2 phase. This mechanism of action was accurately demonstrated by cell cycle analysis, confirming the ability of **2** and **41** to block cells in G2 phase and thus explaining the marked reduction of cell proliferation recorded with the Eg5 inhibitors series. Furthermore, cell cycle analysis revealed a stronger capability of **41** in reducing S phase due to its capability to simultaneously increase the percentage of cells in G2 phase and reduce those in G1 phase. In addition, it can be argued that cell proliferation inhibition, provoked by HSD administration, is related to the possibility to induce a cell cycle arrest on G1-S phase, in accordance with the literature [40]. This mechanism of action could be responsible for a strengthening in **K858** activity. When it is administered in combination with HSD, an additional reduction in cell proliferation and cell percentage in S phase are recorded, thus allowing one to hypothesize a strengthened effect between the two molecules.

Angiogenic event is a key phase during tumour growth and development. The tumoral mass requires oxygen and nutrients supplied by an efficient blood system; at the same time, a good blood system can deliver anti-cancer drugs directly to the mass; thus, antiangiogenic molecules are often considered key strategies in cancer therapy [45]. Exertier et al. (2013) found that an Eg5 inhibition in in vivo zebrafish and chick embryos models induced vascular defect suppressing tumour angiogenesis and proliferation and inhibiting in vitro endothelial cells proliferation and migration, thus shedding light for the first time on new possible roles for Eg5 inhibitors in cancer therapy [46].

The PI3K-Akt pathway is highly involved in angiogenesis and plays a crucial role in gastric carcinoma [47] in which Akt appears to be overexpressed [48], and the recruitment of this signalling cascade is necessary for the spreading of scirrhous gastric carcinoma [49]. We took into consideration the molecules involved in this pathway finding that all Eg5 inhibitors are able to counteract the recruitment of PI3K-Akt signalling downregulating PI3K expression. This finding is strongly supported by the p-Akt/Akt ratio level which exactly shows the same trend of PI3K expression thus confirming the role of Akt in some cancer models in which the silencing of Eg5 resulted in Akt downregulation [18]. Conversely, PI3K levels do not appear considerably influenced by HSD combination with Eg5 inhibitors since, being Akt a downstream target of the pathway, further signalling molecules can contribute to Akt recruitment. HSD also showed a marked capability in modulating the PI3K/Akt signalling, and this appeared even more noticeable if combined with **2**, thus ameliorating the performance of Eg5 inhibitor and confirming the ability of Hesperetin, the aglycone of HSD, to inhibit vascular formation through inhibition of PI3K-Akt pathway [50].

VEGF-A is one of the most important growth factors involved in angiogenesis; being overexpressed in 40–90% of gastric carcinoma cases and it is considered a prognostic biomarker [51]. VEGF interaction with VEGFR-2 receptor can activate the PI3K-Akt pathway [52,53], and at the same time, the activation of PI3K-Akt signalling cascade into cancer cells can regulate VEGF secretion [54]. PI3K-Akt capability to regulate VEGF was definitely confirmed by our results, as VEGF secretion level accurately follows the PI3K-Akt trend. This suggested that Eg5 inhibitors and HSD may modulate the PI3K-Akt-VEGF pathway, thus affecting the molecular signalling at the basis of the angiogenetic process. In addition, the high VEGF expression, especially after 48 h of treatment, does not seem to correspond to increased VEGF secretion levels. Thus, even if the protein expression machinery is launched, the treatment with Eg5 inhibitors alone or in combination with HSD counteracts the passages between protein expression and its final secretion within the medium.

Furthermore, the reduction of VEGF expression and secretion in the AGS cell line treated with all Eg5 inhibitors in monotherapy could be also attributable to a consistent reduction of Erk 1/2 activation measured in our experimental model. VEGF is a downstream molecule of Erk 1/2 and in several tumours of the digestive tract, the suppression of Erk 1/2activation can result in a VEGF downregulation [18,55,56]. Again, after 72 h of treatment, the combination of HSD with **41** and **K858** revealed an appreciable improvement of Eg5 inhibitors in controlling Erk1/2 pathway activation, thus confirming the capability of polyphenols in suppressing vascular formation by blocking Erk activity [50].

To complete the analysis of the molecular signalling underlying the angiogenesis process, ANGPT2 gene expression level was evaluated. It is well-known that ANGPT2 is an antagonist of blood endothelial cells formation through the inhibition of ANGPT1 activity on Tie2 receptor and its expression levels are low in physiological conditions [57]. Surprisingly, in our experimental model, **41** alone induced a consistent ANGPT2 gene expression, more than its parent compound **K858**, thus definitely contrasting one of the angiogenic molecular signalling, whereas **2** capability in promotingANGPT2 expression, appeared appreciable when combined with HSD thus confirming that the two molecules intervened and were able to control different molecular cascades. Considering that the combination HSD+**2** seems to be the most effective in negatively modulating angiogenic molecular signalling and that, as widely reported, a tumoral progression implies an upstream cell migration [58], AGS cells migration, after HSD+**2** treatment, was taken into consideration. Actually, the addition of HSD to **2** definitely reduces cell migration, thus representing a promising tool to counteract tumour evolution in an in vitro model.

## 4. Materials and Methods

### 4.1. Chemistry

**K858**, **2,** and **41** were synthesized, purified (≥96% purity, checked by means of HPLC-UV analyses), and characterized as previously described [36]. Compounds were stored at −20 °C in a 50 mM DMSO stock solution till the biological evaluation. HSD was purchased from Sigma-Aldrich (Milan, Italy) as a white powder with purity ≥97.0% (as determined by HPLC analysis) and dissolved in DMSO to a final 45 mM stock solution.

### 4.2. In Silico Experiments

The crystal structure of Eg5 in complex with ADP and **K858** (PDB ID entry: 6G6Z) and its primary sequence (UniProt code: P52732) were obtained from the PDB and UniProt databases, respectively. To avoid errors during docking simulation, missing side chains and steric clashes in the PDB file were adjusted through protein structure optimization, using MODELLER v.9.3 [59], while loop regions were reconstructed from the Loops in Protein database.

The molecular structures of synthesized compounds were designed with Chemdraw 20 and Marvin Sketch tools. Energy minimization was performed with the USCF Chimera 1.16 package [60] using 1000 steepest descent steps and 10 conjugate gradient steps.

Ligands and receptor for docking calculation were prepared with OpenBabel [61] script by MGL-Tools [62], then for each compound, the molecular docking study was carried out by Autodock/VinaXB software [63]. The grid box was set to 10 Å × 8 Å × 8 Å with a grid space value of 1 Å. The binding box was centered at the binding site of **K858**. Autodock-Vina XB program was used setting default parameters and a maximum of 10 poses per ligand were collected. The results were analysed using PyMOL v2.0. Protein-Ligand Interaction Profiler (PLIP) was used to detect the interactions network all the sets were by default [64,65]. Docking energy scores, as the calculated binding free energies (ΔG), were found −8.1 and −9.1 kcal/mol for compounds **2** and **41**, respectively.

### 4.3. Cell Culture

AGS human gastric adenocarcinoma cell line (ECACC 89090402, Sigma Aldrich, Milan, Italy) was cultured in Ham’s F12 medium supplemented with 10% of foetal bovine serum (FBS), 1% of penicillin/streptomycin, and 1% of l-glutamine (all purchased from EuroClone, Milan, Italy). Cell culture was kept in a humidified atmosphere with 5% CO_2_ at 37 °C.

### 4.4. MTT Assay

AGS cell line was seeded into a 96-well tissue culture plate at a density of 8000 cells/well. Cell metabolic activity was measured by the MTT (3-(4,5-dimethylthiazol-2-yl)-2,5-diphenyltetrazolium bromide) test (Sigma-Aldrich, Milan, Italy), performed after 24, 48, and 72 h of treatment with HSD alone at 9, 18, 36, 72, 144, 180, and 225 µM and after 48 and 72 h of treatment with HSD 180 µM in combination with **2** (1 µM), **41** (5 µM), and **K858** (1 µM). At the established experimental times, the medium was discarded and replaced with a fresh one added with 0.5 mg/mL of MTT. After 5 h of incubation, it was substituted with DMSO for 30 min. The coloured solution obtained by formazan crystal salt dissolution, formed through the capability of viable cells to reduce MTT into formazan, was read at 540 nm using a GO microplate spectrophotometer (Thermo Fisher Scientific, Waltham, MA, USA). The percentage of metabolically active cells was obtained through a normalization with values of cells treated with DMSO (set as 100%).

### 4.5. Cell Cycle Analysis

AGS cells were seeded in 6-well plates with a cell density of 175,000 cells/well. After 48 h of treatment as previously described, the cells were trypsinized, collected by centrifugation, and fixed in cold ethanol 70% *v/v*. After an overnight fixation at 4 °C, cells were washed once with cold phosphate buffer saline (PBS) and centrifuged at 4000 rpm for 10 min. After having discarded supernatants, each sample was incubated with 300 µL of a staining solution containing PBS, Rnase 100 µg/mL (stock solution 10 mg/mL in 10 mM sodium acetate buffer, pH 7.40) and propidium iodide (PI) 10 µg/mL (stock solution 1 mg/mL in water) (all purchased from Sigma Aldrich, MI, USA) and maintained overnight at 4 °C in the dark. The PI fluorescence was detected by a flow cytometer equipped with a 488 nm laser (CytoFlex flow cytometer, Beckman Coulter, CA, USA) in the FL-3 channel. At least 10,000 events/sample were collected and analysed with the CytExpert Software (Beckman Coulter, CA, USA), and the percentages of cells in the G1, S, or G2 phase of the cell cycle were calculated using the ModFit LT™ Software (Verity Software House, Topsham, ME, USA).

### 4.6. Protein Extraction and Western Blot Analysis

AGS cells were seeded in a 6-well plate at a density of 250,000 cells/well and treated with HSD, **2**, **41,** and **K858** alone and HSD in combination with the Eg5 inhibitors. At the established time points, cells were trypsinized and collected by centrifugation at 1200× *g*. Pellets were washed in ice-cold PBS and resuspended in RIPA buffer with freshly added inhibitors (PMSF 100 µg/mL, Aprotinin 10 µg/mL, Leupeptin 50 µg/mL and Sodium Orthovanadate 1 mM, all purchased by Sigma-Aldrich, Milan, Italy). Cell lysates were collected as the whole-cell fraction by centrifugation at 15,000× *g* for 15 min in the cold. The protein concentration was measured through a bicinchoninic acid assay (QuantiPro™ BCA Assay kit for 0.5–30 µg/mL protein, Sigma-Aldrich, Milan, Italy) following the manufacturer’s indications.

For each sample, 20 µg of lysate were separated on a 10% (SDS)-polyacrylamide gel by electrophoresis and transferred to a nitrocellulose membrane. The membrane was blocked in 5% non-fat milk, 10 mmol/L Tris-HCl pH 7.50, 100 mmol/L NaCl, 0.1% (*v/v*) Tween-20 and probed with anti-Akt mouse monoclonal (1:1000) (purchased by Origene Technologies, Rockville, MD, USA), anti-Phospho-Akt (p-Akt) rabbit polyclonal (1:1000), anti-phosphoinositide 3-kinase (PI3K) rabbit polyclonal (1:1000), anti-phospho-p44/42 MAPK (p-Erk1/2) rabbit monoclonal (1:1000), anti-p44/42 MAPK (Erk1/2) rabbit polyclonal (1:1000) (all purchased from Cell Signalling Technology, Danvers, MA, USA), anti-Vascular Endothelial-derived Growth Factor (VEGF) rabbit polyclonal (1:200), and anti-Tubulin mouse monoclonal (1:10,000) (all purchased by Sigma-Aldrich, St. Louis, MO, USA) antibodies. After overnight incubation with primary antibodies at 4 °C under gently shaking, the membrane was incubated with specific IgG horseradish peroxidase (HRP)-conjugated secondary antibodies (Calbiochem, Darmstadt, Germany). Immunoreactive bands were revealed using the ECL detection system (LiteAblot Extend Chemiluminescent Substrate, EuroClone S.p.a., Milan, Italy) and underwent densitometry. Densitometric values, expressed as Integrated Optical Intensity, were estimated using a ChemiDoc™ XRS system and the QuantiOne 1-D analysis software (BIORAD, Richmond, CA, USA). Values obtained were normalized based on densitometric values of internal Tubulin.

### 4.7. ELISA Analysis of VEGF Secretion

After 48 and 72 h of treatment, AGS cells supernatants were collected from a 6-well plate used for cell lysate. VEGF ELISA kit (Enzo Life Sciences, Farmingdale, NY, USA) was used to perform a quantitative determination (pg/mL) of human VEGF released in the medium. Then, 100 µL of samples were loaded into a VEGF microtiter plate and incubated at room temperature (RT) for 60 min under shaking. After 4 washings with specific wash buffer, 100 µL of diluted VEGF detector antibody was added into each well and probed at RT for 30 min under shaking. By adding VEGF Conjugate to each well and then a TBM solution, the plate was read at 450 nm with a GO microplate spectrophotometer (Thermo Fisher Scientific, Waltham, MA, USA). VEGF pg/mL values were obtained by using a standard curve generated with specific standards, provided by the manufacturer.

### 4.8. RNA Extraction

After 24 h of treatment, cells were trypsinized and collected by centrifugation, after washing with PBS. To extract RNA PureLink^®^ RNA Mini Kit (Life Technologies, Carlsbad, CA, USA) was applied. Then 300 µL of supplied lysis buffer, added with 1% of 2-mercaptoethanol and 300 µL of ethanol 70% *v/v*; were added to each cell pellet; samples were transferred into the Spin Cartridge for RNA extraction and purification. After washing with Wash Buffer supplied by the kit, samples were probed for 15 min with 80 µL of DNase mixture (On-column PureLink^®^ DNase Treatment, Life Technologies) to remove contaminating DNA. RNA extracted from each sample was eluted in 30 µL of Nuclease-Free Water. RNA concentration (ng/µL) was determined through Qubit^®^ RNA BR Assay Kits (Life Technologies, Carlsbad, CA, USA).

### 4.9. Reverse Transcription (RT) and Real-Time RT-Polymerase Chain Reaction (Real-Time RT-PCR)

High-capacity cDNA Reverse Transcription Kit (Life Technologies, Carlsbad, CA, USA) was used to reverse transcribe 1 µg of RNA in a reaction volume of 20 µL. Reactions were incubated in a thermal cycler at 25 °C for 10 min, 37 °C for 2 h, and 85 °C for 5 min. The run method consisted of the following steps: 50 °C for 2 min, 95 °C for 10 min, 95 °C for 15 s, 60 °C for 1 min. Steps 2 and 3 were repeated for 40 cycles.

For all the examined mRNAs, gene expression was determined by quantitative PCR using PowerUp^TM^ SYBR^TM^ Green Master Mix (2×) (Thermo Fisher Scientific, Waltham, MA, USA). Each amplification reaction was performed in a MicroAmp^®^ Optical 96-weel Reaction Plate (Life Technologies, Carlsbad, CA, USA) in a reaction volume of 20 µL made up of 10 µL of SYBR Green, 1 µM of each primer (stock solution 100 µM), 10 ng of cDNA and Nuclease-Free Water. Primer sequences used are reported in Table 1.

The run method consisted of 50 °C for 2 min, 95 °C for 2 min, 40 cycles of amplification at 95 °C for 15 s and 60 °C for 1 min in QuantStudio 3 (Thermo Fisher Scientific, Waltham, MA, USA). QuantStudio™ Design & Analysis Software v1.5.1 (Thermo Fisher Scientific, Waltham, MA, USA) was used to elaborate gene expression data. The authenticity of the PCR products was verified by melt curve analysis. Each gene expression value was normalized to the expression level of GAPDH. The fold changes of the investigated genes were expressed in relation to the level of GAPDH of 24 h. The comparative 2^−ΔΔCt^ method was used to quantify the relative abundance of mRNA (relative quantification).

### 4.10. Wound Healing

AGS cells were seeded in a 6-well plate and when they reached 60–70% confluence cells monolayer was treated with **2**, HSD, and HSD+**2**. Each monolayer was scraped with a p200 pipet tip. Images were taken after 0, 6 and 24 h of treatment with an inverted light microscope Leica Dmi1 (Leica Cambridge Ltd., Cambridge, UK) equipped with a camera Leica MC120 HD (Leica Cambridge Ltd., Cambridge, UK) for computerized images.

### 4.11. Statistical Analysis

Statistics were performed using *t*-test and one-way analysis of variance (ANOVA) followed by Tukey’s multiple comparison test by means of the Prism 5.0 software (GraphPad, San Diego, CA, USA). The results are the mean values ± SD. Values of *p* ≤ 0.05 were considered statistically significant.

## 5. Conclusions

In summary, the newly synthesized Eg5 inhibitors **2** and **41** show a promising profile in antitumoral activity with a major extent in gastric adenocarcinoma. In particular, the results presented in this study confirm a valuable antiproliferative effect. In addition, these compounds seem to play a pivotal role in controlling the molecular machinery underlying the angiogenic event even better than the parent compound **K858**. Furthermore, the combination of HSD with **K858** and **41** markedly emphasizes the antiproliferative effect, whereas the combination of HSD with **2** underlines an effective control of the signalling involved in angiogenesis occurrence, leading to hypothesize that Eg5 inhibitors, even if showing a similar chemical structure, intervene and regulate different molecular cascades, thus representing an innovative strategy in the development of anti-cancer therapeutic protocols. Further investigations are needed to deepenthe dual effect of Eg5 inhibitors and evaluate if it could be related to interaction with off-target proteins.

## Figures and Tables

**Table 1:** Primer sequences for quantitative PCR.

Gene	Sequence (5′ to 3′)
GAPDH-FW	GGGTGTGAACCATGAGAAGTA
GAPDH-RW	ACTGTGGTCATGAGTCCTTC
ANGPT2-FW	ACCTGTTGAACCAAACAGCG
ANGPT2-RW	GTCGAGAGGGAGTGTTCCAAG

## Data Availability

The datasets generated and analyzed in the current study are contained within the article or provided in the Supporting Information file. Details are available from the corresponding authors on reasonable request.

## Supplementary Materials

The following are available online. Figure S1. Histogram view of the distribution of datasets found for search queries “Human Kinesin” (blue) and “Human Kinesin Inhibitors” (red) per year (1986–2022) in PubMed Database, Figure S2. Phylogenetic dendrogram for human Kinesins, Figure S3. The targets–components analysis performed on the STITCH platform. Lines are related to confidence view: stronger associations are represented by thicker lines. Protein-protein interactions are shown in grey, chemical-protein interactions in green, and interactions between chemicals in red, Figure S4. The protein–protein association analyses performed on the STRING platform. On the right, both functional and physical protein associations; on the left, only physical protein association (proteins are involved in a physical complex). Lines are related to confidence view: stronger associations are represented by thicker lines. Table S1. One-way analysis of variance (ANOVA) followed by Tukey’s multiple comparison test performed by means of the Prism 5.0 software (GraphPad, San Diego, CA, USA). References [66,67,68,69,70,71,72,73] are cited in the supplementary materials.

## Sample Availability

Samples of compounds **2** and **41** are available from the authors.
