# Supplementary material for: Negative Modulation of the Angiogenic Cascade Induced by Allosteric Kinesin Eg5 Inhibitors in a Gastric Adenocarcinoma In Vitro Model
Source: Molecules. 2022 Jan 31;27(3):957. doi: 10.3390/molecules27030957 (PMC8840372; doi:10.3390/molecules27030957)
Supplement: Supplementary file 1 [file molecules-27-00957-s001.zip › molecules-1519844-supplementary.pdf]

# Supporting Information

## Negative Modulation of the Angiogenic Cascade Induced by Allosteric Kinesin Eg5 Inhibitors in a Gastric Adenocarcinoma In Vitro Model

**Alessia Ricci [1], Marialucia Gallorini [1], Donatella Del Bufalo [2], Amelia Cataldi [1], Ilaria D'Agostino [1], Simone Carradori [1,†] and Susi Zara [1,†,*]**

[1] Department of Pharmacy, University "G. d'Annunzio" of Chieti-Pescara, , 66100 Chieti, Italy; alessia.ricci@unich.it (A.R.); marialucia.gallorini@unich.it (M.G.); amelia.cataldi@unich.it (A.C.); ilaria.dagostino1@unich.it (I.D.); simone.carradori@unich.it (S.C.)

[2] Preclinical Models and New Therapeutic Agents Unit, IRCCS Regina Elena National Cancer Institute, Rome, Italy; donatella.delbufalo@ifo.it (D.D.B)

* Correspondence: susi.zara@unich.it; Tel.: +39-0871-3554507

† The authors equally contributed to this work.

## List of Contents

Dataset for Kinesins topics in PubMed Database

Phylogenetic analysis

Protein-chemicals interaction network

Protein-protein association network

ANOVA and Tukey's multiple comparison for data shown in Figures 5, 6, 7, 8, 9, and 12.

## Dataset for Kinesins topics in PubMed Database

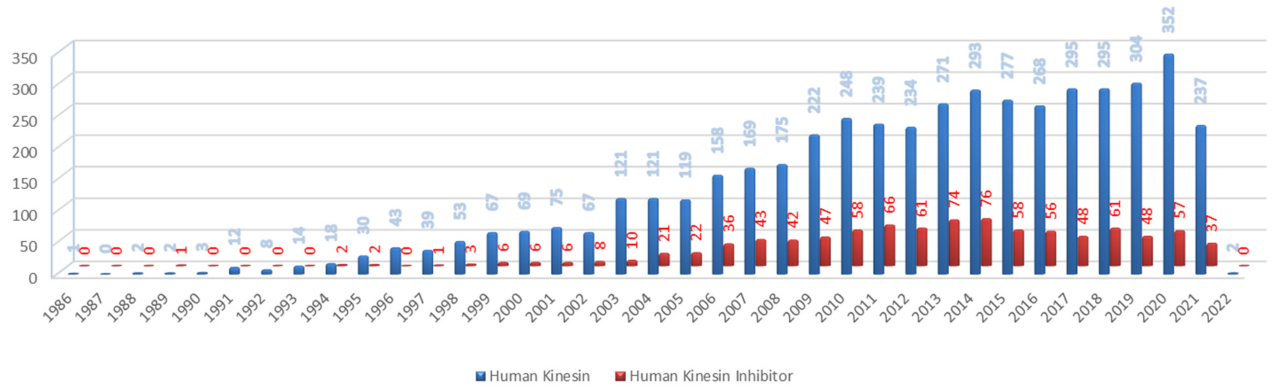


**Figure S1.** Histogram view of the distribution of datasets found for search queries «Human Kinesin» (blue) and «Human Kinesin Inhibitors» (red) per year (1986-2022) in PubMed Database.

## Phylogenetic analysis

A phylogenetic tree, reported in Figure S2, was conducted on the NGphylogeny.fr free service (https://ngphylogeny.fr, accessed on 6 January 2022) [1,2] through the "one click workflow" tool and FastME2.0 program to gain the "distances" among phylogenies based on the balanced minimum evolution approach. Sequences were selected from UniProt Database by searching the following keywords "name:kinesin-like protein" and "organism: *Homo sapiens* (Human) [9606]", filtering only reviewed sequences.

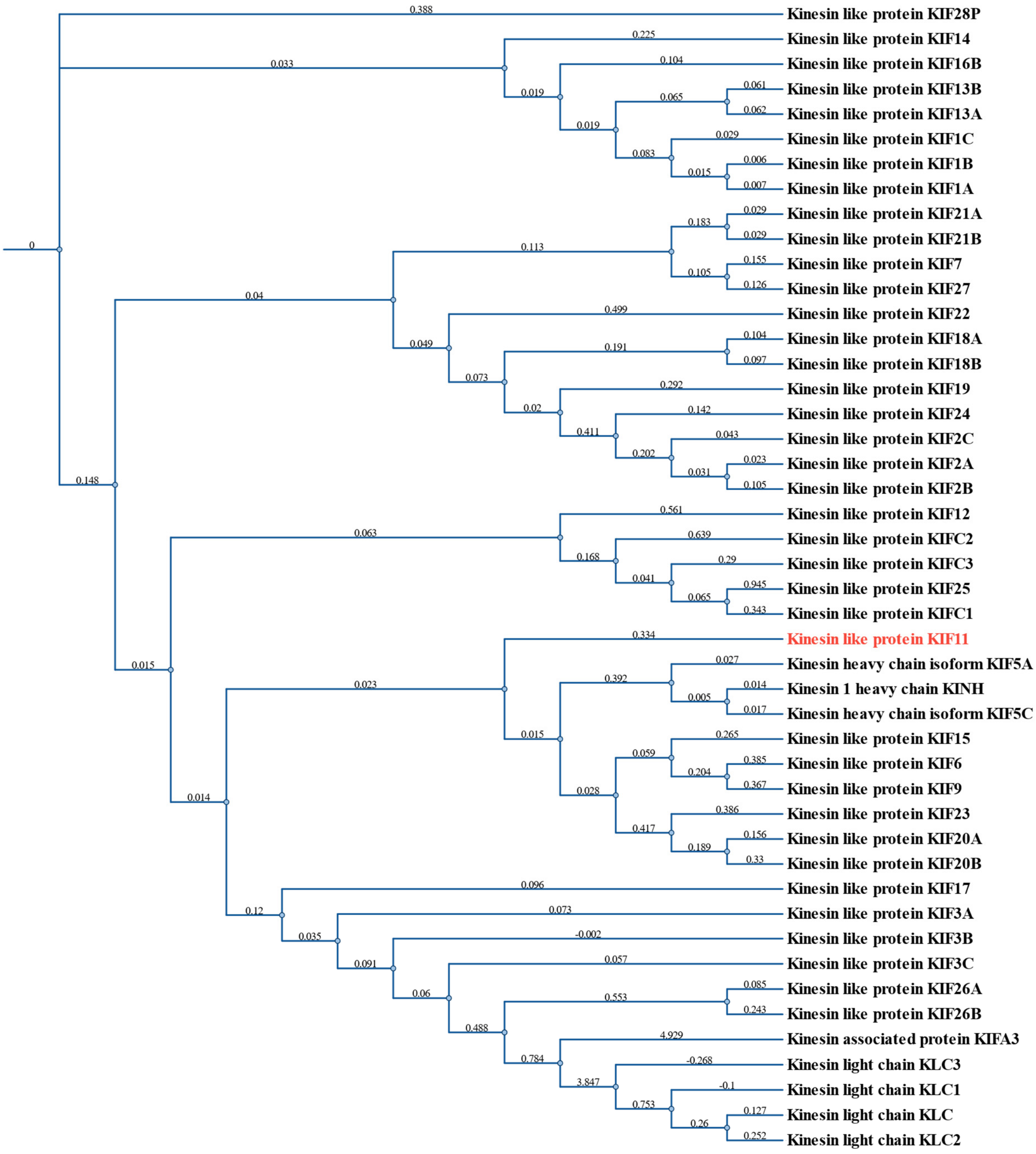


**Figure S2.** Phylogenetic dendrogram for human Kinesins.

The evolutionary model was determined automatically using NGphylogeny.fr free service. The tree branches are drawn to scale, which represents the number of differences between sequences through evolution. In red, human KIF11. The figure was prepared by means of the Interactive Tree Of Life (iTOL) (https://itol.embl.de/, accessed on 06 January 2022) [3].

## Protein-chemicals interaction network

The bioinformatics software platform STITCH (http://stitch.embl.de) was used to predict direct interactions of FIK-11 with compounds.

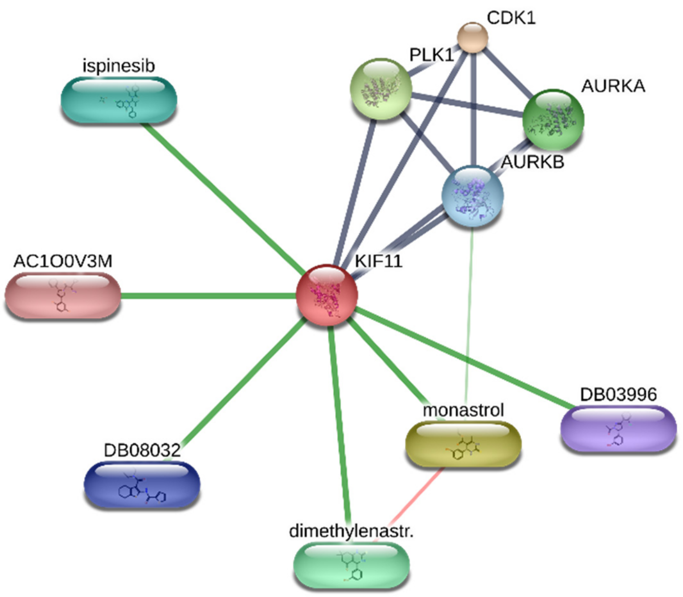


**Figure S3.** The targets–components analysis performed on the STITCH platform. Lines are related to confidence view: stronger associations are represented by thicker lines. Protein-protein interactions are shown in grey, chemical-protein interactions in green and interactions between chemicals in red.

Proteins are CDK1, Cyclin-dependent kinase 1; PLK1, Polo-like kinase 1; AURKA, Aurora kinase A; AURKB, Aurora kinase B.

## Protein-protein association network

The bioinformatics software platform STRING (http://string.embl.de) was used to predict direct interactions of FIK-11 with other proteins.

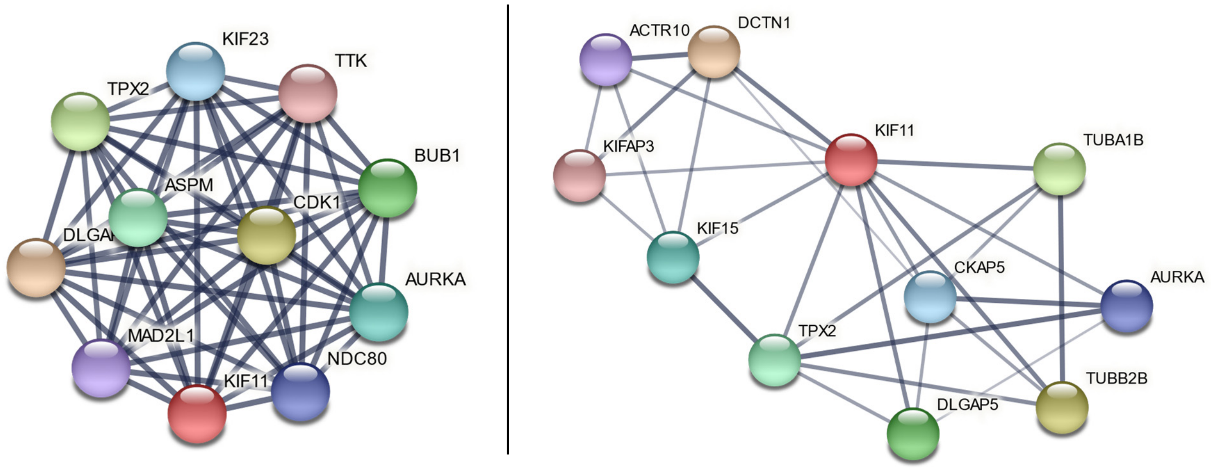


**Figure S4.** The protein-protein association analyses performed on the STRING platform. On the right, both functional and physical protein associations; on the left, only physical protein association (proteins are involved in a physical complex). Lines are related to confidence view: stronger associations are represented by thicker lines.

Proteins are DLGAP5, Disks large-associated protein 5 [4]; CDK1, cyclin-dependent kinase 1; TPX2, microtubule nucleation factor [5]; BUB1, Mitotic checkpoint serine/threonine-protein kinase; ASPM, Abnormal spindle-like microcephaly-associated protein; AURKA, Aurora kinase A [6]; KIF23, Kinesin-like protein; NDC80, Kinetochore protein NDC80 homolog; MAD2L1, Mitotic spindle assembly checkpoint protein MAD2A; TTK, Serine/threonine-protein kinase ttk/mps1; DCTN1, Dynactin subunit 1 [7]; TUBB2B, Tubulin beta-2B chain; TUBA1B, Tubulin alpha-1B chain; KIF15, Kinesin-like protein [8]; CKAP5, Cytoskeleton-associated protein 5; ACTR10, Actin related protein 10 homolog; KIFAP3, Kinesin-associated protein 3.

## ANOVA and Tukey's multiple comparison for data shown in Figures 5, 6, 7, 8, 9, and 12

**Table S1.** One-way analysis of variance (ANOVA) followed by Tukey's multiple comparison test performed by means of the Prism 5.0 software (GraphPad, San Diego, CA, USA).

| **ANOVA Analysis of Figure 5** | | | | | |
|---|---|---|---|---|---|
| | | | | | |
| **Table Analysed** | **G1 Phase-48 h** | | | | |
| | | | | | |
| ***One-way analysis of variance*** | | | | | |
| P value | < 0.0001 | | | | |
| P value summary | *** | | | | |
| Are means signif. different? (P < 0.05) | Yes | | | | |
| Number of groups | 8 | | | | |
| F | 20.99 | | | | |
| R squared | 0.913 | | | | |
| | | | | | |
| ***ANOVA Table*** | ***SS*** | ***df*** | ***MS*** | | |
| Treatment (between columns) | 2328 | 7 | 332.6 | | |
| Residual (within columns) | 221.9 | 14 | 15.85 | | |
| Total | 2550 | 21 | | | |
| | | | | | |
| ***Tukey's Multiple Comparison Test*** | ***Mean Diff.*** | ***q*** | ***Significant? P < 0.05?*** | ***Summary*** | ***95% CI of diff*** |
| **DMSO vs 41** | 31.51 | 12.26 | Yes | *** | 18.68 to 44.33 |

| **Table Analysed** | **S Phase-48 h** | | | | |
|---|---|---|---|---|---|
| | | | | | |
| ***One-way analysis of variance*** | | | | | |
| P value | < 0.0001 | | | | |
| P value summary | *** | | | | |
| Are means signif. different? (P < 0.05) | Yes | | | | |
| Number of groups | 8 | | | | |
| F | 25.51 | | | | |
| R squared | 0.9273 | | | | |

| | | | | | |
|---|---|---|---|---|---|
| | | | | | |
| *ANOVA Table* | *SS* | *df* | *MS* | | |
| Treatment (between columns) | 392.5 | 7 | 56.07 | | |
| Residual (within columns) | 30.77 | 14 | 2.198 | | |
| Total | 423.3 | 21 | | | |

| *Tukey's Multiple Comparison Test* | *Mean Diff.* | *q* | *Significant? P < 0.05?* | *Summary* | *95% CI of diff* |
|---|---|---|---|---|---|
| **DMSO vs HSD** | 8.02 | 9.37 | Yes | *** | 3.749 to 12.29 |
| **DMSO vs 2** | 10.37 | 12.12 | Yes | *** | 6.099 to 14.64 |
| **DMSO vs 41** | 9.68 | 10.12 | Yes | *** | 4.905 to 14.46 |
| **K858 vs HSD+K858** | 5.903 | 6.897 | Yes | ** | 1.632 to 10.17 |
| | | | | | |
| **Table Analysed** | **G2 Phase-48 h** | | | | |
| | | | | | |
| *One-way analysis of variance* | | | | | |
| P value | < 0.0001 | | | | |
| P value summary | *** | | | | |
| Are means signif. different? (P < 0.05) | Yes | | | | |
| Number of groups | 8 | | | | |
| F | 16.62 | | | | |
| R squared | 0.8926 | | | | |
| | | | | | |
| *ANOVA Table* | *SS* | *df* | *MS* | | |
| Treatment (between columns) | 3353 | 7 | 478.9 | | |
| Residual (within columns) | 403.4 | 14 | 28.81 | | |
| Total | 3756 | 21 | | | |
| | | | | | |
| *Tukey's Multiple Comparison Test* | *Mean Diff.* | *q* | *Significant? P < 0.05?* | *Summary* | *95% CI of diff* |
| **DMSO vs 41** | -41.19 | 11.89 | Yes | *** | -58.47 to -23.90 |

| ANOVA Analysis of Figure 6 | | | | | |
|---|---|---|---|---|---|
| | | | | | |
| **Table Analysed** | **PI3K protein expression 48 h (Fig. 6B)** | | | | |
| | | | | | |
| *One-way analysis of variance* | | | | | |
| P value | < 0.0001 | | | | |
| P value summary | *** | | | | |
| Are means signif. different? (P < 0.05) | Yes | | | | |
| Number of groups | 8 | | | | |
| F | 36.06 | | | | |
| R squared | 0.9404 | | | | |
| | | | | | |
| *ANOVA Table* | *SS* | *df* | *MS* | | |
| Treatment (between columns) | 0.01639 | 7 | 0.002341 | | |
| Residual (within columns) | 0.001039 | 16 | 0.00006492 | | |
| Total | 0.01743 | 23 | | | |
| | | | | | |
| *Tukey's Multiple Comparison Test* | *Mean Diff.* | *q* | *Significant? P < 0.05?* | *Summary* | *95% CI of diff* |
| **DMSO vs 2** | 0.02947 | 6.335 | Yes | ** | 0.006692 to 0.05225 |
| **DMSO vs 41** | 0.06465 | 13.9 | Yes | *** | 0.04187 to 0.08743 |
| **DMSO vs K858** | 0.06726 | 14.46 | Yes | *** | 0.04448 to 0.09004 |
| | | | | | |
| **Table Analysed** | **PI3K protein expression 72 h (Fig. 6B)** | | | | |
| | | | | | |
| *One-way analysis of variance* | | | | | |
| P value | < 0.0001 | | | | |
| P value summary | *** | | | | |
| Are means signif. different? (P < 0.05) | Yes | | | | |

| | | | | | |
|---|---|---|---|---|---|
| Number of groups | 8 | | | | |
| F | 60.02 | | | | |
| R squared | 0.9633 | | | | |
| | | | | | |
| *ANOVA Table* | *SS* | *df* | *MS* | | |
| Treatment (between columns) | 0.05414 | 7 | 0.007734 | | |
| Residual (within columns) | 0.002062 | 16 | 0.0001289 | | |
| Total | 0.0562 | 23 | | | |
| | | | | | |
| *Tukey's Multiple Comparison Test* | *Mean Diff.* | *q* | *Significant? P < 0.05?* | *Summary* | *95% CI of diff* |
| **DMSO vs HSD** | 0.07024 | 10.72 | Yes | *** | 0.03815 to 0.1023 |
| **DMSO vs 2** | 0.1224 | 18.68 | Yes | *** | 0.09034 to 0.1545 |
| **DMSO vs 41** | 0.1149 | 17.53 | Yes | *** | 0.08276 to 0.1469 |
| **DMSO vs K858** | 0.1571 | 23.97 | Yes | *** | 0.1250 to 0.1892 |
| | | | | | |
| **Table Analysed** | **p-Akt/Akt protein expression 48 h (Fig. 6C)** | | | | |
| | | | | | |
| *One-way analysis of variance* | | | | | |
| P value | < 0.0001 | | | | |
| P value summary | *** | | | | |
| Are means signif. different? (P < 0.05) | Yes | | | | |
| Number of groups | 8 | | | | |
| F | 230.1 | | | | |
| R squared | 0.9902 | | | | |
| | | | | | |
| *ANOVA Table* | *SS* | *df* | *MS* | | |
| Treatment (between columns) | 0.87 | 7 | 0.1243 | | |
| Residual (within columns) | 0.008642 | 16 | 0.0005401 | | |
| Total | 0.8787 | 23 | | | |

| | | | | | |
|---|---|---|---|---|---|
| | | | | | |
| *Tukey's Multiple Comparison Test* | *Mean Diff.* | *q* | *Significant? P < 0.05?* | *Summary* | *95% CI of diff* |
| **DMSO vs 2** | 0.1949 | 14.53 | Yes | *** | 0.1292 to 0.2606 |
| **DMSO vs 41** | 0.4006 | 29.85 | Yes | *** | 0.3349 to 0.4663 |
| **DMSO vs K858** | 0.3442 | 25.65 | Yes | *** | 0.2785 to 0.4099 |
| | | | | | |
| **Table Analysed** | **p-Akt/Akt protein expression 72 h (Fig. 6C)** | | | | |
| | | | | | |
| *One-way analysis of variance* | | | | | |
| P value | < 0.0001 | | | | |
| P value summary | *** | | | | |
| Are means signif. different? (P < 0.05) | Yes | | | | |
| Number of groups | 8 | | | | |
| F | 544.6 | | | | |
| R squared | 0.9958 | | | | |
| | | | | | |
| *ANOVA Table* | *SS* | *df* | *MS* | | |
| Treatment (between columns) | 3.401 | 7 | 0.4859 | | |
| Residual (within columns) | 0.01428 | 16 | 0.0008922 | | |
| Total | 3.415 | 23 | | | |
| | | | | | |
| *Tukey's Multiple Comparison Test* | *Mean Diff.* | *q* | *Significant? P < 0.05?* | *Summary* | *95% CI of diff* |
| **DMSO vs HSD** | 0.5713 | 33.13 | Yes | *** | 0.4868 to 0.6557 |
| **DMSO vs 2** | 0.761 | 44.13 | Yes | *** | 0.6766 to 0.8455 |
| **DMSO vs 41** | 1.168 | 67.74 | Yes | *** | 1.084 to 1.253 |
| **DMSO vs K858** | 1.06 | 61.49 | Yes | *** | 0.9760 to 1.145 |
| **2 vs HSD+2** | 0.2231 | 12.94 | Yes | *** | 0.1387 to 0.3076 |

| ANOVA Analysis of Figure 7 | | | | | |
|---|---|---|---|---|---|
| | | | | | |
| **Table Analysed** | **VEGF protein expression 48 h (Fig. 7B)** | | | | |
| | | | | | |
| ***One-way analysis of variance*** | | | | | |
| P value | < 0.0001 | | | | |
| P value summary | *** | | | | |
| Are means signif. different? (P < 0.05) | Yes | | | | |
| Number of groups | 8 | | | | |
| F | 74.89 | | | | |
| R squared | 0.9704 | | | | |
| | | | | | |
| ***ANOVA Table*** | ***SS*** | ***df*** | ***MS*** | | |
| Treatment (between columns) | 0.02415 | 7 | 0.00345 | | |
| Residual (within columns) | 0.000737 | 16 | 0.00004606 | | |
| Total | 0.02488 | 23 | | | |
| | | | | | |
| ***Tukey's Multiple Comparison Test*** | ***Mean Diff.*** | ***q*** | ***Significant? P < 0.05?*** | ***Summary*** | ***95% CI of diff*** |
| **DMSO vs HSD** | 0.02875 | 7.338 | Yes | ** | 0.009564 to 0.04794 |
| **DMSO vs 41** | 0.06718 | 17.14 | Yes | *** | 0.04799 to 0.08637 |
| | | | | | |
| **Table Analysed** | **VEGF protein expression 72 h (Fig. 7B)** | | | | |
| | | | | | |
| ***One-way analysis of variance*** | | | | | |
| P value | < 0.0001 | | | | |
| P value summary | *** | | | | |
| Are means signif. different? (P < 0.05) | Yes | | | | |
| Number of groups | 8 | | | | |

| | | | | | |
|---|---|---|---|---|---|
| F | 253.5 | | | | |
| R squared | 0.9911 | | | | |
| | | | | | |
| *ANOVA Table* | *SS* | *df* | *MS* | | |
| Treatment (between columns) | 0.05936 | 7 | 0.008479 | | |
| Residual (within columns) | 0.0005352 | 16 | 0.00003345 | | |
| Total | 0.05989 | 23 | | | |
| | | | | | |
| *Tukey's Multiple Comparison Test* | *Mean Diff.* | *q* | *Significant? P < 0.05?* | *Summary* | *95% CI of diff* |
| **DMSO vs 2** | 0.07029 | 21.05 | Yes | *** | 0.05394 to 0.08665 |
| **DMSO vs 41** | 0.07851 | 23.51 | Yes | *** | 0.06215 to 0.09486 |
| **DMSO vs K858** | 0.1186 | 35.51 | Yes | *** | 0.1022 to 0.1349 |
| **2 vs HSD+2** | 0.02893 | 8.664 | Yes | *** | 0.01258 to 0.04528 |
| **41 vs HSD+41** | 0.04408 | 13.2 | Yes | *** | 0.02773 to 0.06043 |

| **ANOVA Analysis of Figure 8** | | | | | |
|---|---|---|---|---|---|
| | | | | | |
| **Table Analysed** | **VEGF secretion 48 h (Fig. 8A)** | | | | |
| | | | | | |
| *One-way analysis of variance* | | | | | |
| P value | < 0.0001 | | | | |
| P value summary | *** | | | | |
| Are means signif. different? (P < 0.05) | Yes | | | | |
| Number of groups | 8 | | | | |
| F | 102.7 | | | | |
| R squared | 0.989 | | | | |
| | | | | | |
| *ANOVA Table* | *SS* | *df* | *MS* | | |
| Treatment (between columns) | 11910000 | 7 | 1702000 | | |
| Residual (within columns) | 132600 | 8 | 16570 | | |
| Total | 12040000 | 15 | | | |
| | | | | | |
| *Tukey's Multiple Comparison Test* | *Mean Diff.* | *q* | *Significant? P < 0.05?* | *Summary* | *95% CI of diff* |
| **DMSO vs 2** | 1219 | 13.39 | Yes | *** | 709.7 to 1729 |
| **DMSO vs 41** | 2237 | 24.57 | Yes | *** | 1727 to 2746 |
| **DMSO vs K858** | 1733 | 19.04 | Yes | *** | 1223 to 2242 |
| | | | | | |
| **Table Analysed** | **VEGF secretion 72 h (Fig. 8B)** | | | | |
| | | | | | |
| *One-way analysis of variance* | | | | | |
| P value | < 0.0001 | | | | |
| P value summary | *** | | | | |
| Are means signif. different? (P < 0.05) | Yes | | | | |
| Number of groups | 8 | | | | |
| F | 114.1 | | | | |
| R squared | 0.9901 | | | | |

| | | | | | |
|---|---|---|---|---|---|
| | | | | | |
| *ANOVA Table* | *SS* | *df* | *MS* | | |
| Treatment (between columns) | 64470000 | 7 | 9210000 | | |
| Residual (within columns) | 646000 | 8 | 80750 | | |
| Total | 65110000 | 15 | | | |
| | | | | | |
| *Tukey's Multiple Comparison Test* | *Mean Diff.* | *q* | *Significant? P < 0.05?* | *Summary* | *95% CI of diff* |
| **DMSO vs HSD** | 1276 | 6.351 | Yes | * | 151.5 to 2401 |
| **DMSO vs 2** | 2333 | 11.61 | Yes | *** | 1208 to 3458 |
| **DMSO vs 41** | 5905 | 29.39 | Yes | *** | 4780 to 7029 |
| **DMSO vs K858** | 4281 | 21.3 | Yes | *** | 3156 to 5405 |

| ANOVA Analysis of Figure 9 | | | | | |
|---|---|---|---|---|---|
| | | | | | |
| **Table Analysed** | **p-Erk/Erk protein expression 48h (Fig. 9B)** | | | | |
| | | | | | |
| *One-way analysis of variance* | | | | | |
| P value | < 0.0001 | | | | |
| P value summary | *** | | | | |
| Are means signif. different? (P < 0.05) | Yes | | | | |
| Number of groups | 8 | | | | |
| F | 228.5 | | | | |
| R squared | 0.9901 | | | | |
| | | | | | |
| *ANOVA Table* | *SS* | *df* | *MS* | | |
| Treatment (between columns) | 0.04112 | 7 | 0.005874 | | |
| Residual (within columns) | 0.0004112 | 16 | 0.0000257 | | |
| Total | 0.04153 | 23 | | | |
| | | | | | |
| *Tukey's Multiple Comparison Test* | *Mean Diff.* | *q* | *Significant? P < 0.05?* | *Summary* | *95% CI of diff* |
| **DMSO vs HSD** | 0.05397 | 18.44 | Yes | *** | 0.03963 to 0.06830 |
| **DMSO vs 2** | 0.07823 | 26.73 | Yes | *** | 0.06389 to 0.09256 |
| **DMSO vs 41** | 0.1372 | 46.89 | Yes | *** | 0.1229 to 0.1516 |
| **DMSO vs K858** | 0.1333 | 45.53 | Yes | *** | 0.1189 to 0.1476 |
| | | | | | |
| **Table Analysed** | **p-Erk/Erk protein expression 72h (Fig. 9B)** | | | | |
| | | | | | |
| *One-way analysis of variance* | | | | | |
| P value | < 0.0001 | | | | |

| | | | | | |
|---|---|---|---|---|---|
| P value summary | *** | | | | |
| Are means signif. different? (P < 0.05) | Yes | | | | |
| Number of groups | 8 | | | | |
| F | 984.7 | | | | |
| R squared | 0.9977 | | | | |
| | | | | | |
| ***ANOVA Table*** | ***SS*** | ***df*** | ***MS*** | | |
| Treatment (between columns) | 0.1829 | 7 | 0.02614 | | |
| Residual (within columns) | 0.0004246 | 16 | 0.00002654 | | |
| Total | 0.1834 | 23 | | | |
| | | | | | |
| ***Tukey's Multiple Comparison Test*** | ***Mean Diff.*** | ***q*** | ***Significant? P < 0.05?*** | ***Summary*** | ***95% CI of diff*** |
| **DMSO vs 2** | 0.1293 | 43.46 | Yes | *** | 0.1147 to 0.1438 |
| **DMSO vs 41** | 0.1908 | 64.16 | Yes | *** | 0.1763 to 0.2054 |
| **DMSO vs K858** | 0.1864 | 62.66 | Yes | *** | 0.1718 to 0.2009 |
| **41 vs HSD+41** | 0.06067 | 20.4 | Yes | *** | 0.04610 to 0.07523 |
| **K858 vs HSD+K858** | 0.02993 | 10.06 | Yes | *** | 0.01536 to 0.04449 |

| ANOVA Analysis of Figure 12 | | | | | |
|---|---|---|---|---|---|
| | | | | | |
| **Table Analysed** | **% Cut width reduction 6 h (Fig. 12)** | | | | |
| | | | | | |
| *One-way analysis of variance* | | | | | |
| P value | 0.0599 | | | | |
| P value summary | ns | | | | |
| Are means signif. different? (P < 0.05) | No | | | | |
| Number of groups | 4 | | | | |
| F | 3.751 | | | | |
| R squared | 0.5845 | | | | |
| | | | | | |
| *ANOVA Table* | *SS* | *df* | *MS* | | |
| Treatment (between columns) | 154.8 | 3 | 51.61 | | |
| Residual (within columns) | 110.1 | 8 | 13.76 | | |
| Total | 264.9 | 11 | | | |
| | | | | | |
| *Tukey's Multiple Comparison Test* | *Mean Diff.* | *q* | *Significant? P < 0.05?* | *Summary* | *95% CI of diff* |
| **DMSO vs HSD+2** | 9.733 | 4.545 | Yes | * | 0.03375 to 19.43 |
| | | | | | |
| **Table Analysed** | **% Cut width reduction 24 h (Fig. 12)** | | | | |
| | | | | | |
| *One-way analysis of variance* | | | | | |
| P value | < 0.0001 | | | | |
| P value summary | *** | | | | |
| Are means signif. different? (P < 0.05) | Yes | | | | |
| Number of groups | 4 | | | | |
| F | 46.34 | | | | |
| R squared | 0.9456 | | | | |
| | | | | | |

| | | | | | |
|---|---|---|---|---|---|
| *ANOVA Table* | *SS* | *df* | *MS* | | |
| Treatment (between columns) | 4044 | 3 | 1348 | | |
| Residual (within columns) | 232.7 | 8 | 29.09 | | |
| Total | 4277 | 11 | | | |
| | | | | | |
| *Tukey's Multiple Comparison Test* | *Mean Diff.* | *q* | *Significant? P < 0.05?* | *Summary* | *95% CI of diff* |
| **DMSO vs HSD** | 43.57 | 13.99 | Yes | *** | 29.46 to 57.67 |
| **DMSO vs 2** | 24.9 | 7.996 | Yes | ** | 10.80 to 39.01 |
| **DMSO vs HSD+2** | 45.7 | 14.68 | Yes | *** | 31.60 to 59.81 |
| **2 vs HSD+2** | 20.8 | 6.68 | Yes | ** | 6.698 to 34.91 |
| | | | | | |
| **Table Analysed** | **% Cut width reduction 72 h (Fig. 12)** | | | | |
| | | | | | |
| *One-way analysis of variance* | | | | | |
| P value | < 0.0001 | | | | |
| P value summary | *** | | | | |
| Are means signif. different? (P < 0.05) | Yes | | | | |
| Number of groups | 4 | | | | |
| F | 50.84 | | | | |
| R squared | 0.9502 | | | | |
| | | | | | |
| *ANOVA Table* | *SS* | *df* | *MS* | | |
| Treatment (between columns) | 3373 | 3 | 1124 | | |
| Residual (within columns) | 176.9 | 8 | 22.11 | | |
| Total | 3549 | 11 | | | |
| | | | | | |
| *Tukey's Multiple Comparison Test* | *Mean Diff.* | *q* | *Significant? P < 0.05?* | *Summary* | *95% CI of diff* |
| **DMSO vs HSD** | 36.97 | 13.62 | Yes | *** | 24.68 to 49.27 |
| **DMSO vs 2** | 24.16 | 8.898 | Yes | ** | 11.86 to 36.45 |
| **DMSO vs HSD+2** | 44.02 | 16.21 | Yes | *** | 31.72 to 56.32 |
| **2 vs HSD+2** | 19.86 | 7.317 | Yes | ** | 7.569 to 32.16 |

## References


1. Dereeper, A.; Guignon, V.; Blanc, G.; Audic, S.; Buffet, S.; Chevenet, F.; Dufayard, J.F.; Guindon, S.; Lefort, V.; Lescot, M.; Claverie, J.M.; Gascuel, O. Phylogeny.fr: robust phylogenetic analysis for the non-specialist. *Nucleic Acids Res.* 2008, 36, W465–W469. doi: 10.1093/nar/gkn180.
2. Lemoine, F.; Correia, D.; Lefort, V.; Doppelt-Azeroual, O.; Mareuil, F.; Cohen-Boulakia, S.; Gascuel, O. NGPhylogeny.fr: new generation phylogenetic services for non-specialists. *Nucleic Acids Res.* 2019, 47, W260–W265. doi: 10.1093/nar/gkz303.
3. Letunic, I.; Bork, P. Interactive Tree Of Life (iTOL) v5: an online tool for phylogenetic tree display and annotation. *Nucleic Acids Res.* 2021, 49, W293–W296. doi:10.1093/nar/gkab301.
4. Liu, J.; Liu, Z.; Zhang, X.; Gong, T.; Yao, D. Examination of the expression and prognostic significance of DLGAPs in gastric cancer using the TCGA database and bioinformatic analysis. *Mol. Med. Rep.* 2018, 18, 5621–5629. doi:10.3892/mmr.2018.9574.
5. Huang, C.; Hu, C.G.; Ning, Z.K.; Huang, J.; Zhu, Z.M. Identification of key genes controlling cancer stem cell characteristics in gastric cancer. *World J Gastrointest Surg.* 2020, 12, 442–459. doi:10.4240/wjgs.v12.i11.442.
6. Dar, A.A.; Zaika, A.; Piazuelo, M.B.; Correa, P.; Koyama, T.; Belkhiri, A.; Washington, K.; Castells, A.; Pera, M.; El-Rifai, W. Frequent overexpression of Aurora Kinase A in upper gastrointestinal adenocarcinomas correlates with potent antiapoptotic functions. *Cancer* 2008, 112, 1688–1698. doi: 10.1002/cncr.23371.
7. Zhang, J.; Goel, A.; Zhu, L. Identification of Novel Alternative Splicing Events Associated With Tumorigenesis, Protein Modification, and Immune Microenvironment in Early-Onset Gastric Cancer. *Front Oncol.* 2021, 11 640272. doi: 10.3389/fonc.2021.640272.
8. Ding, L.; Li, B.; Yu, X.; Li, Z.; Li, X.; Dang, S.; Lv, Q.; Wei, J.; Sun, H.; Chen, H.; Liu, M.; Li, G. KIF15 facilitates gastric cancer via enhancing proliferation, inhibiting apoptosis, and predict poor prognosis. *Cancer Cell Int.* 2020, 20, 125. doi:10.1186/s12935-020-01199-7.
